# Reduced breastfeeding rates among obese mothers: a review of contributing factors, clinical considerations and future directions

**DOI:** 10.1186/s13006-015-0046-5

**Published:** 2015-07-01

**Authors:** Jennie Bever Babendure, Elizabeth Reifsnider, Elnora Mendias, Michael W. Moramarco, Yolanda R. Davila

**Affiliations:** College of Nursing and Health Innovation, Arizona State University, Phoenix, AZ 85004 USA; University of Texas Medical Branch at Galveston, School of Nursing, Galveston, TX 77555 USA

**Keywords:** Breastfeeding rates, Breastfeeding initiation, Maternal obesity, Breastfeeding intervention

## Abstract

Maternal obesity is associated with significantly lower rates of breastfeeding initiation, duration and exclusivity. Increasing rates of obesity among reproductive-age women has prompted the need to carefully examine factors contributing to lower breastfeeding rates in this population. Recent research has demonstrated a significant impact of breastfeeding to reduce the risk of obesity in both mothers and their children. This article presents a review of research literature from three databases covering the years 1995 to 2014 using the search terms of breastfeeding and maternal obesity. We reviewed the existing research on contributing factors to lower breastfeeding rates among obese women, and our findings can guide the development of promising avenues to increase breastfeeding among a vulnerable population. The key findings concerned factors impacting initiation and early breastfeeding, factors impacting later breastfeeding and exclusivity, interventions to increase breastfeeding in obese women, and clinical considerations. The factors impacting early breastfeeding include mechanical factors and delayed onset of lactogenesis II and we have critically analyzed the potential contributors to these factors. The factors impacting later breastfeeding and exclusivity include hormonal imbalances, psychosocial factors, and mammary hypoplasia. Several recent interventions have sought to increase breastfeeding duration in obese women with varying levels of success and we have presented the strengths and weaknesses of these clinical trials. Clinical considerations include specific techniques that have been found to improve breastfeeding incidence and duration in obese women. Many obese women do not obtain the health benefits of exclusive breastfeeding and their children are more likely to also be overweight or obese if they are not breastfed. Further research is needed into the physiological basis for decreased breastfeeding among obese women along with effective interventions supported by rigorous clinical research to advance the care of obese reproductive age women and their children.

## Introduction

Data indicate that overweight and obesity among reproductive aged women has increased in the last decade and nearly 60 % of reproductive aged women in the US are overweight or obese. This contributes to the 31 % rate of overweight and obesity in US children aged 2–19 years [1–3].

Maternal pre-pregnancy obesity (Body mass index, BMI > 30) is associated with up to 13 % lower rates of breastfeeding initiation, and 20 % decreased likelihood of any breastfeeding at six months postpartum [4–15]. Breastfeeding has a dose-response-like effect to reduce the risk of childhood obesity by as much as 32 %, and significantly reduces the risk of obesity-associated co- morbidities such as diabetes, high blood pressure and elevated cholesterol in both mothers and their breastfed children [16–23]. Yet obese mothers are among the least likely to breastfeed as long or as exclusively as recommended [17, 19]. Dramatic differences exist in breastfeeding rates between countries and cultures; however there appears to be a significant effect of maternal obesity to reduce both breastfeeding initiation and duration independent of nation of study [24–34]. In the United States, studies have demonstrated between 7 and 13 % decreases in breastfeeding incidence among obese women [35–37]. Similarly, the Longitudinal Study of Australian Children [14] demonstrated that obese Australian women were 8 % less likely to initiate breastfeeding, and that increasing body mass index (BMI) over 30 had a dose-response-like effect to reduce breastfeeding incidence. Among those who initiated breastfeeding, obese mothers were 7 % less likely to continue breastfeeding to one week and almost 13 % less likely to be breastfeeding at six months [14]. A 2007 study in Danish women also demonstrated a dose-response-type of relationship between increasing BMI > 30 and lower incidence of breastfeeding [38]. Finding this association in societies that are supportive of breastfeeding (Denmark and Australia) suggests that socio-cultural issues may not be a major cause of decreased breastfeeding initiation in obese women [39, 40]. Even among those with known medical conditions (diabetes, pre-eclampsia) associated with lower breastfeeding rates, maternal obesity further increases the risk of early breastfeeding cessation [41–45].

Our objectives for this review were to summarize the existing research on potential causes of reduced breastfeeding incidence, exclusivity and duration in obese women, present the results of the existing breastfeeding interventions in this population, and discuss clinical considerations of relevance and future directions for research.

## Methods

**Fig. 1:**
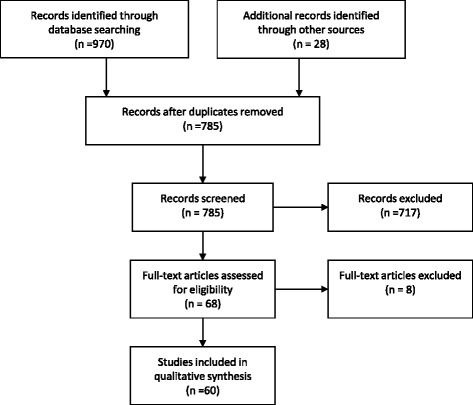
PRISMA 2009 flow diagram used when selecting articles for this review

We performed a literature search of Web of Science, PubMed (Medline), and the Cochrane database with the search terms “breastfeeding” and “maternal obesity” from 1995 to August 2014, restricted to English (PubMed, Web of Science) and humans (PubMed) (see Fig. 1) [46]. Criteria for inclusion were peer reviewed original research reporting on breastfeeding initiation, exclusivity or duration, or factors related to breastfeeding initiation, exclusivity or duration in obese mothers (BMI ≥ 30) of term infants (37–42 weeks gestation). Authors screened abstracts with these criteria, and then accessed full text articles. From the full text articles, five meeting abstracts were removed, as were two articles with definitions of obesity that included groups categorized by the World Health Organization as overweight (BMI ≥26), and one article focusing on weight retention in obese mothers in relation to breastfeeding. The references of chosen articles and the authors’ libraries were also hand searched for relevant studies. Articles reporting only on obesity as a risk factor for non-initiation or early cessation of breastfeeding are incorporated into the article introduction. Those reporting on interventions or factors relating to breastfeeding outcomes in obese women are included in the body of the review.

## Review

### Factors impacting early breastfeeding

#### Mechanical factors/edema

Mechanical factors, such as additional body tissue, larger areolas and larger breasts that reduce lap area have often been cited as an impediment to breastfeeding in obese women [6, 7, 9, 40, 47, 48]. Despite the frequency of this suggestion, we found no published studies documenting a significant impact of mechanical factors on breastfeeding in obese mothers. There is evidence, however, that obese women are more likely to experience significant postpartum edema [12].

This can result in pooling of the fluid load in the breasts, flattening the nipples and making latch more difficult. Beyond this, some, although not all, obese women may be more likely to have larger breasts, which can make traditional breastfeeding positions more challenging [47].

#### Delayed onset of lactogenesis II

Lactogenesis II, or the onset of copious milk production, is triggered by transcription of prolactin-responsive genes following removal of placental progesterone [49]. For most mothers, lactogenesis II occurs within 72 h postpartum. Studies have demonstrated that maternal obesity is associated with delays in the onset of lactogenesis II (DOL) which can reduce the mother’s confidence that her milk is sufficient for her child, lead to early introduction of breast milk substitutes, and result in early cessation of breastfeeding [48]. A small observational study in a Swiss hospital found that obese mothers were more likely to use “breastfeeding aids” such as bottles, cups and maltodextrin supplementation [50], and this observation is consistent with the findings of larger studies indicating that obese mothers are less likely to be exclusively breastfeeding at hospital discharge, even among those who intend to breastfeed exclusively [51].

Research suggests that DOL in obese women may be due to several factors. Obesity increases the risk of significant postpartum edema, which is independently associated with DOL [12]. Obese mothers also have an increased incidence of longer and dysfunctional labor and higher rates of cesarean birth, which are also associated with DOL [52]. Moynihan et al. have suggested that the increase in dysfunctional labor and cesarean birth in obese mothers may be linked to a leptin-dependent blunting of oxytocin stimulated muscle contraction [53]. Leptin, an adipokine, is secreted by adipose tissue, and leptin levels increase with increasing BMI. In vitro, leptin inhibits oxytocin’s effect on muscle contractions, potentially contributing to increases in labor dysfunction observed in obese women [53]. As oxytocin is also necessary for the milk ejection reflex, elevated leptin levels in obese women may negatively influence milk availability. In addition, Rasmussen and Kjolhede showed in 2004 that obese women have reduced baseline levels of prolactin in the first 48 h postpartum, and reduced suckling-induced release of prolactin at 2–7 days postpartum, which may reduce the rate of milk synthesis during this time [54, 55]. Interestingly, Kitsantas and Pawloski found that obese mothers with no medical or labor complications were as likely to initiate breastfeeding as non-obese mothers, although they were 11 % more likely to stop breastfeeding each month thereafter [13]. As lactogenesis II generally occurs 24–72 h after birth and breastfeeding initiation, this finding is not inconsistent with observed delays in the onset of lactogenesis in obese mothers.

Several studies now suggest that insulin is required for lactogenesis II and an imbalance in insulin may directly influence the timing of lactogenesis II. Obese women have a less steep decline in insulin concentrations from the end of pregnancy to the initiation of lactation, perhaps leading to less glucose available for milk synthesis [56]. Nommsen-Rivers, Dolan, and Huang recently found that the insulin/glucose ratio measured at 26 weeks of gestation could predict the timing of lactogenesis II [57]. Similarly, RNA sequencing of the human milk fat layer demonstrated increased expression of protein tyrosine phosphatase receptor type F, a phosphatase known to down-regulate insulin signaling in a group of mothers with DOL [58].

Each of these factors may contribute to DOL in obese women.

### Factors impacting longer breastfeeding duration and exclusivity in obese women

#### Androgens

In addition to alterations in prolactin and insulin, free androgens also increase with increasing BMI in women [59]. A recent study by Carlsen et al. found a negative correlation between mid-pregnancy androgen levels and breastfeeding duration at 3 and 6 months [60]. Some data also indicate that polycystic ovarian syndrome (PCOS) may play a role in reducing breastfeeding duration and exclusivity in obese women [61, 62]. PCOS is associated with elevated androgens, metabolic abnormalities and hypothyroidism, and PCOS often occurs alongside overweight or obesity [63]. A 2008 study by Vanky et al. found that in the first month of lactation mothers with PCOS were less likely to be exclusively breastfeeding [64]. The authors suggested this difference might be due to slightly elevated 3^rd^ trimester levels of the androgen Dehydroepiandrosterone (DHEA) in mothers with PCOS [64].

#### Thyroid dysfunction

Obesity and overweight may result from subclinical and overt hypothyroidism, the symptoms of which (fatigue, weight gain, hair loss) may be dismissed as normal postpartum complaints [65]. Animal models have demonstrated the requirement for levothyroxine (T4) and liothyronine (T3) in initiation and maintenance of lactation, and human mothers with suboptimal levels may have lower milk supply and reduced oxytocin release in response to suckling [66].

Treatment with levothyroxine to bring maternal T4 and thyroid stimulating hormone (TSH) levels within normal range generally alleviates the problems hypothyroidism presents to lactation [67]. Multiple sources now recommend that serum TSH levels between 0.5 and 2.5 are optimal for pregnancy and perhaps lactation as well [68, 69].

#### Psychosocial factors

Several studies have linked obesity to psychosocial factors that independently reduce breastfeeding initiation, exclusivity and duration. In their 2014 study of 2824 American participants in the Infant Feeding Practices Study II (IFPSII), Hauff, Leonard and Rasmussen found that obese women demonstrated reduced confidence in their ability to reach their own breastfeeding goals (*p* < 0.0001), fewer close friends and relatives who had breastfed (*p* < 0.0001), and lower social influence to breastfeed (*p* < 0.02) [70]. Obese mothers in this study did not have reduced intention to breastfeed, but did have lower odds of ever breastfeeding, and were at greater risk of early breastfeeding discontinuation [70]. This finding agrees with that of Kronberg, Vaeth and Rasmussen, who found maternal obesity to be associated with lower maternal self-efficacy and a higher rate of early breastfeeding cessation in a study of 1597 Danish mothers [71]. In their study of 233 overweight and obese women, Hauff and Demerath reported obese women with body image concerns had a lower median duration of breastfeeding than normal weight women without body image concerns (38.6 weeks v 48.9 weeks, respectively; p = 0.01) [40]. The authors also reported the probability of obese women to breastfeed at six months at 66 % in comparison to 80 % for normal weight women [40]. They suggest that discomfort with their bodies may cause obese women to be hesitant to breastfeed [40]. By contrast, Zanardo et al. found that Italian obese mothers in a case-control study had higher scores on Eating Disorders Inventory (EDI-2) measures such as body dissatisfaction (*p* < 0.001), but were significantly less likely than non-obese mothers to stop breastfeeding before six months [71]. Another study with the same population found that the obese mothers were also more likely to report body image dissatisfaction [72]. Foster et al. reported in 1996 that women who intended to bottle feed had higher levels of body dissatisfaction (median 47.5 v 38.5, p = 0.004) and higher expressed concerns about shape/weight (median 1.05 v 0.29, p = 0.02) than those who intended to breastfeed, however, breastfeeding intention was not correlated with BMI in this small study [73]. Krause et al. found that unrealistic expectation of the effect of breastfeeding to reduce maternal weight may negatively impact breastfeeding duration among overweight and obese mothers [74].

Although most studies have found an independent association between maternal obesity and reduced breastfeeding incidence and duration, some have found sociodemographic characteristics to be more significant. In a prospective cohort study of 718 women in Hershey Pennsylvania, Bartok et al. found that obese mothers had a shorter duration of providing breast milk and an earlier introduction of formula than non-obese mothers. However, when the authors controlled for confounding variables, the association with BMI disappeared, and only education, marital status, planned breastfeeding duration, and rating of breastfeeding importance significantly impacted breastfeeding duration [75]. In addition, studies have not found maternal obesity to have a significant impact on breastfeeding incidence and duration in African American women [8, 37]. This finding may be related to low baseline levels of breastfeeding incidence in the African American population in general [76].

#### Mammary hypoplasia/insufficient glandular tissue

Some evidence from research in dairy cows and mice suggests that obesity in early life may negatively impact breast glandular development [77, 78]. This may put obese mothers at risk of insufficient glandular tissue and/or mammary hypoplasia [78, 79]. For example, a study of dairy cows fed a high energy diet during puberty demonstrated increased mammary adipose tissue and reduced mammary epithelium in adulthood [77]. Obese mice also show reductions in mammary gland development and the milk proteins β casein, whey acidic protein, and αlactalbumin, which are essential for milk production [78]. Classic markers for mammary hypoplasia in humans are wide intramammary space, breast asymmetry, stretch marks, and little or no breast growth in pregnancy [79]. However, insufficient glandular tissue may also be present without these physical characteristics [80]. In agreement with findings in animals, obese mothers are more likely to report characteristics consistent with insufficient milk supply than are non-obese mothers [81]. Mok et al. found that only 60 % of obese women perceived their milk supply to be adequate at one month postpartum compared with 94 % of normal weight controls [81]. Obese women are almost twice as likely to indicate they stopped breastfeeding due to insufficient milk (24 % vs. 13 %), and women with higher BMI report insufficient milk earlier than women with BMI in the normal range [82, 83]. In a study of breast milk expression using a breast pump or hand expression in the first two months postpartum, obese women were more likely to try to express milk, but less likely to be successful [84]. The significantly lower rate of exclusive breastfeeding among obese women also suggests obese mothers may feel they do not make sufficient breast milk to feed their babies, and need to supplement the infant’s diet to provide adequate calories [10, 81–83, 85]. Interestingly, in an analysis of IFPSII data, Leonard et al. found that the tendency of women with higher BMI to deliver heavier infants actually underestimates the magnitude of the negative impact of maternal obesity on breastfeeding duration, as heavier infants are breastfed for longer duration in the general population [86]. This may partially explain the unusual results of a retrospective cohort study in Ontario which found that mothers with Class III obesity (n = 249, BMI ≥ 40), who were significantly more likely to give birth to a baby that was large for gestational age (LGA), were as likely to initiate breastfeeding and be breastfeeding at hospital discharge as mothers with normal BMI (n = 446, BMI 18.5–24.9) [87].

### Interventions to increase breastfeeding in obese women

In contrast to the many interventions conducted to increase breastfeeding in the general population, only four published interventions have focused on increasing breastfeeding duration and exclusivity specifically in obese women (see Table 1). The first two, published in 2011 by Rasmussen et al., were conducted in parallel based in rural Bassett Hospital in New York, called the Bassett Improving Breastfeeding Studies (BIBS) 1 and 2 [88]. The BIBS1 study protocol called for the intervention group (n = 19) to receive three telephone calls by one of three International Board Certified Lactation Consultants (IBCLC), one call prenatally and then at 48 and 72 h to educate, assist with and encourage breastfeeding. Mothers in the control group (n = 20) were also given a less detailed prenatal call. BIBS1 was poorly executed in that only 11 mothers in each of the control and intervention groups received the appropriate interventions.

**Table 1:** Interventions to increase breastfeeding in obese mothers

Authors, year of publication, country	Study population	Intervention	Control	Breastfeeding outcomes	Child health outcomes
Rasmussen et al. 2011 [88] Rural New York, USA (BIBS1)	BMI > 29 carrying singleton infants recruited at ≤ 35 weeks gestation, delivering at ≥ 37 wks gestation in Rural Bassett Hospital	3 telephone calls by one of 3 IBCLCs. One call prenatally and then at 48 and 72 h to educate, assist with and encourage breastfeeding.	1 prenatal telephone call (less detailed)	EBF median: support 3.4wks (25^th^–75^th^ % 0.7–8.4) control 8.1wks (2.1–13.1)	Not assessed
				Any BF median: support 8.6wks (3.9–13.0), control 12.6wks (9.1–13.5)	
	n = 40				
Rasmussen et al. 2011 [88] Rural New York, USA (BIBS2)	BMI > 29 carrying singleton infants recruited at ≥ 35 weeks gestation, delivering at ≥ 37 wks gestation in Rural Bassett Hospital	Mothers given manual or multiuser electric breastpump and instructed to pump for 10 min after each of 5 breastfeeding sessions each 24 h for 5 days or until their milk came in.	Usual care. No breastpump or instructions given	EBF median: manual pump 2.3wks (0.4–4.4), electric pump 0.7wks (0.1–2.7), control 4.4wks (1.1–9.4)	Not assessed
				Any BF median: manual pump 13.4wks (2.1–36.0), electric pump 4wks (2.4–8.4), control 26.6wks (9.4–44.6) p < 0.004 for pumping groups	
	n = 39				
Chapman et al. 2013 [89] Connecticut, USA	BMI ≥ 27 carrying singleton infants recruited at ≤ 36 weeks gestation, from prenatal Baby Friendly Hospital clinic, income < 185 % of the federal poverty level with telephone access. Infants ≥ 36 weeks’ gestation, birth weight ≥2.5 kg and ≤ 3.9 kg, 1 and 5 min Apgar scores of ≥ 6, and no NICU admission.	3 prenatal visits, daily in-hospital support, phone access, up to 11 postpartum home visits from specialized obesity-trained breastfeeding peer counselors. Home visits tentatively scheduled 3 per week in 1^st^ week, 2 per week in weeks 2–4, 1 per week in weeks 5 and 6. Phone call between 2 and 3 months. Large breastfeeding sling, single electric breastpump if separated for work/school. Mothers had work phone number of peer counselors.	3 prenatal visits, daily in hospital support and up to 7 home visits from Breastfeeding Heritage peer counselors. Mothers had work phone number of peer counselors.	Any BF at 2 weeks: AOR 3.76 (95 % CI: 1.07, 13.22)	Odds of hospitalization in first 6 months after birth: AOR 0.24 (95 % CI: 0.07, 0.86)
				≥50 % of feedings as breast milk at 2 weeks: AOR 4.47 (95 % CI: 1.38, 14.5)	
	N = 206				
Carlsen et al. 2013 [90] Denmark	BMI ≥ 30 delivering healthy singleton infants at term participating in prenatal weight gain reduction (TOPS) study in Denmark recruited at < 48 h postpartum	Minimum of 9 telephone consultations by a single IBCLC if continuing to breastfeed. First call in first week postpartum, 2 more calls in first month, every 2 weeks until 8 weeks, and monthly until 6 months. Extra calls for specific difficulties, mothers had study IBCLC phone number	Usual care, including contact with a breastfeeding supportive pediatric nurse within 1 week of birth, and standard breastfeeding support at study hospital	EBF median: Support 120d (14-142d)	Days of exclusive breastfeeding inversely associated with:
				Control 41d (3-133d) p = 0.003	Infant weight at 6 months
				Any BF median: Support 184d (92–185d) Control 108d (16–185d) p = 0.002	β = 4.39 g/day, (95 % CI: −0.66, −8.11 p = 0.021)
	n = 207				
				EBF 3 months: AOR 2.45 (95 % CI: 1.36, 4.41 p = 0.003)	Infant length at 6 months
				Any BF 6 months: AOR 2.25 (95 % CI: 1.24, 4.08 p = 0.008)	β = 0.012 cm/day (95 % CI: −0.004, 0.02 p = 0.004)

*BF* breastfeeding, *EBF* exclusive breastfeeding, *AOR* adjusted odds ratio, *CI* confidence interval

Baseline data indicated that the intervention and control groups were non-equivalent in BMI at delivery. When adjusted for BMI at delivery, logistic regression found the probability of any breastfeeding at 30 days was significantly less in the intervention group (*p* < 0.04), and any breastfeeding was highly correlated with BMI at delivery at 90 days (*p* < 0.03) [88].

BIBS2 looked at the impact of the provision of a breast pump on breastfeeding duration in a similar population. Intervention group mothers were given a single manual or multiuser double electric breast pump and instructed to pump for 10 min after each of five breastfeeding sessions each 24 h for five days or until their milk came in [88]. When compared with the control group and analyzed as intent to treat, mothers given a pump breastfed for significantly shorter duration [88]. There were significant challenges in this study as well, as many mothers in the control group used a pump, and mothers in the intervention group used their own rather than a study pump [88]. When controlled for type of pump used, there was no difference in breastfeeding duration between mothers who used a manual or an electric pump [88]. Further analysis revealed that BMI at delivery was significantly associated with shorter breastfeeding at the 30 day time point (*p* < 0.03) [88]. When BMI at delivery was added to the logistic regression model, the differences in breastfeeding duration between pumping and control groups were no longer significant [88]. The lack of stratification for obesity and small sample size of both BIBS1 (n = 40) and BIBS2 (n = 39) limit the usefulness of these studies in designing future interventions [88].

From 2006–2009, Chapman et al. conducted a randomized controlled trial using breastfeeding education and support provided both in person and by phone by peer counselors [89]. Two hundred and six low income overweight and obese women (BMI ≥ 27) who delivered in a Baby Friendly Hospital in Connecticut were randomized to either a control group which included three prenatal visits, phone access, daily in hospital support, and up to seven home visits from a breastfeeding peer counselor, or an intervention group which included three prenatal visits, daily in-hospital support, and up to 11 postpartum home visits from a specialized breastfeeding peer counselor who had 20 h of obesity-specific breastfeeding training [89]. The intervention group did not demonstrate a significant increase in breastfeeding duration or exclusivity at 1, 3 or 6 months. Further analysis showed that at two weeks postpartum, mothers in the intervention had greater odds of continuing any breastfeeding (adjusted odds ratio (AOR) 3.76; 95 % confidence interval (CI): 1.07, 13.22), and of giving at least 50 % of feeding as breast milk (AOR 4.47; 95 % CI: 1.38, 14.5) [89]. Infants of intervention mothers were also less likely to be hospitalized during the first six months of age (AOR 0.24; 95 % CI: 0.07, 0.86) [89]. The authors suggest that the limited effects on breastfeeding duration seen with this population may be due to the fact that mothers in the control group were already receiving a significant amount of breastfeeding support [89].

The most recent intervention study provided breastfeeding support via phone calls by an International Board-Certified Lactation Consultant (IBCLC) [90]. The study by Carlsen et al. included 207 obese (BMI ≥ 30) women delivering singleton infants in Hvidovre Hospital in Copenhagen, Denmark who were participating in the Treatment of Obese Pregnant Study (TOPS) to reduce weight gain during pregnancy (gestational weight gain goal < 5 kg) [90].

Mothers in the TOPS study had been assigned to one of 3 groups: 1) exercise alone, 2) exercise and diet, or 3) control [90]. Mothers and their newborns were consecutively recruited into the breastfeeding support intervention without regard to their TOPS study group, allocated into the breastfeeding support intervention or control group [90]. Mothers in the support intervention (n = 105) were offered a minimum of nine telephone consultations by a single IBCLC during the first six months so long as they continued to breastfeed [90]. The initial contact was made within the first week postpartum, and two more contacts were made that month, followed by phone calls every two weeks until eight weeks, then monthly until six months [90]. Extra calls were made for specific difficulties, and all mothers in the intervention group had the direct telephone number to the study IBCLC, available seven days per week [90]. Mothers in the control group (n = 102) had access to usual care, which included contact with a breastfeeding supportive pediatric nurse within one week of birth, and standard breastfeeding support at the study hospital [90]. Both groups were equivalent in BMI prenatally [90]. In contrast to previous studies discussed above, provision of phone call support in this intervention significantly increased breastfeeding duration and exclusivity [90]. Mothers in the intervention group breastfed exclusively for a median of 120 days (25^th^–75^th^ percentiles: 14–142 days) compared with 41 days for control mothers (3–133 days, p = 0.003) [90]. Those given the support intervention also had increased duration of any breastfeeding (median 184 days (92–185 days) compared with those in the control group (median 108 days (16–185 days) p = 0.002) [90]. Support increased the adjusted odds ratio for exclusive breastfeeding at three months (AOR 2.45; 95 % CI: 1.36, 4.41; p = 0.003) and partial breastfeeding at six months (OR 2.25; 95 % CI: 1.24, 4.08; p = 0.008) [90]. An impact on breastfeeding duration was evident even before the phone calls began, indicating that the Hawthorne effect may have played a role [90, 91].

It is of interest that this most recent support intervention [90] resulted in increased breastfeeding duration and exclusivity, in contrast to previous support interventions in obese mothers [89, 90]. As the execution and design of the BIBS studies were flawed, we cannot use them to draw accurate conclusions; however, both Carlsen et al. and Chapman et al. delivered a support intervention to obese mothers [88–90]. Several key differences between these two studies may have impacted the outcome. First, the populations studied were quite dissimilar. Chapman et al. was conducted with a group of low income, > 80 % Latina mothers in the United States, while the Carlsen et al. population was comprised of a group of Danish mothers of unknown socioeconomic status or ethnicity who were already participating in a study to reduce weight gain in pregnancy [89, 90]. The mothers in the Carlsen et al. study had received education about adiposity and healthy lifestyle during pregnancy, and were likely already highly motivated [90]. In addition to differences in ethnicity, socioeconomic status and participation in another lifestyle intervention, the mothers in the Carlsen et al. study were an average of eight years older, and had two years more education than those in the Chapman et al. study [89, 90]. Socioeconomic status, age, and years of education have all been shown to have significant effects on breastfeeding duration and exclusivity [92, 93]. Denmark also provides a full 52 weeks of maternity leave to employed mothers, while the more than 30 % of mothers employed prenatally in the Chapman et al. study may have had access to little or no maternity leave [89, 90]. In the larger (non-obese) population, support interventions have shown an increased treatment effect on exclusive breastfeeding in areas where background breastfeeding rates are high [94]. Breastfeeding initiation, duration, and exclusivity are significantly higher in Denmark than in the US, which may have increased the effectiveness of the support intervention on this outcome measure [95].

The interventions themselves also differed in several ways that may have impacted the study outcome. The Chapman et al. study began prenatally while Carlsen et al. began at three days postpartum [89, 90]. All of the interactions in the Carlsen et al. study were by phone, versus a mixture of phone and in person visits in the Chapman et al. study. This is of interest as a 2012 Cochrane review on breastfeeding support interventions concluded that regular scheduled support was more effective at increasing breastfeeding duration and exclusivity than support that required mothers to ask for help [94]. The design of the Carlsen et al. study included 7 phone calls at regularly scheduled intervals, which may have been easier to implement on a predictable schedule for mothers. In addition, the Carlsen et al. study used a single IBCLC to deliver all contacts, while the Chapman et al. study used multiple peer counselors [89, 90]. The use of a single support person for all mothers rather than multiple contacts with varying communication styles, counseling skills and personal breastfeeding experience may have increased the fidelity of the intervention.

### Clinical considerations

Many clinicians are unaware of research showing that obese women have lower rates of breastfeeding or of the proposed causes [54]. Our efforts to help obese mothers reach their breastfeeding goals are limited both by an incomplete knowledge of the biological factors that may impact breastfeeding success in obese women, and by the small number of interventions targeting this population. In Table 2, we have provided some clinical considerations to be aware of when helping obese mothers to breastfeed.

**Table 2:** Clinical considerations when helping obese mothers to breastfeed successfully

Prenatal	
Obese mothers may benefit from	Rationale
Strategies to limit weight gain in pregnancy	Reduce the risk of preeclampsia, gestational diabetes, LGA baby, and cesarean birth [5].
Discussion of strategies such as doula care and non-pharmacological pain management to reduce the need for labor interventions.	Constant support by a doula or other trained care provider during labor has been shown to shorten the length of labor and reduce the incidence of surgical birth by as much as 40 % in the general population [96, 97].
Intrapartum	
Obese mothers may benefit from	Rationale
Careful evaluation of adequate time to labor	First stage of active labor increases with increasing BMI. Research indicates a need to reevaluate normal labor progression in obese women to establish new guidelines to prevent unnecessary augmentation and surgical intervention [52].
Assistance with non-pharmacological pain management techniques	Long labor and stressful or surgical birth can contribute to DOL [98–100].
Judicious use of pitocin/ IV fluids	Reduce risk of DOL due to postpartum edema [101].
Constant support while laboring	Obese pregnant women have been shown to have higher levels of anxiety and stress, which may contribute to excessive catecholamine levels and reduced uterine contractibility [52].
Early Postpartum	
Obese mothers may benefit from	Rationale
Guidance on how to know baby is getting enough milk	Perception of insufficient milk is the most common reason mothers do not breastfeed as long as desired. This is even more common in obese mothers [81].
Demonstration of multiple feeding positions such as:	Pain is cited as second most common cause of breastfeeding discontinuation. This is even more common in obese mothers [81]. Demonstrating multiple options for positioning allow for better tailoring to mother’s needs, and reduced nipple stress.
Laid-back breastfeeding positions	
Side-lying	
Cradle/cross cradle hold	
Clutch/football/underarm hold	Breastfeeding positions that utilize semi-reclined maternal posture may work particularly well for obese mothers as they utilize mother’s torso to support baby, obviating the need for pillows and breast support. Side-lying positions also provide additional support for breast and baby [102].
Assistance to support large breasts and to better visualize latch	Mothers with large breasts may need additional assistance to visualize latch and breastfeed comfortably [103]. A rolled towel or breast sling to elevate the breast and/or a mirror to visualize nipple and latch may be helpful.
Demonstration of reverse pressure softening around areola to enable deeper latch	Obese mothers are more likely to experience significant postpartum edema, which can temporarily flatten nipples, making latch difficult. Reverse pressure softening, accomplished by holding gentle reverse pressure around the areola toward the chest wall, can be useful in reducing peri-areolar edema [12, 101].
Specific Guidance to supplement only when medically necessary.	Early supplementation is associated with reduced breastfeeding duration and exclusivity, and risk is elevated in children of obese mothers [81].
Use Academy of Breastfeeding Medicine Protocol #3 to verify medical need for supplementation [104]	
Continued support postpartum	Phone support by an IBCLC may increase breastfeeding duration and exclusivity in some populations of obese mothers [90]. Regular phone support, referral to breastfeeding support groups, and skilled in-person care should be a priority in this at-risk population.

### Future direction

Carlsen et al.’s intervention methods led to an increase in breastfeeding duration and exclusivity in a population of older, educated Danish mothers [90]. Future studies should work to reproduce and fine tune this intervention in other populations of obese mothers, including those with lower background breastfeeding rates than those observed in Denmark. As the authors point out, although their intervention did significantly increase rates of breastfeeding in obese women, a full 15 % of the obese women in the intervention group did not successfully establish breastfeeding, and an even larger percentage did not establish exclusive breastfeeding, far below the rates of non-obese Danish women. This suggests that there may be another, perhaps physiological component that is not modifiable by support interventions. Many aspects of lactation are hormonally controlled. As obesity has been shown to alter levels and action of a large number of hormones, tracking the levels and activity of hormones known to impact lactation (such as prolactin, thyroid hormone, TSH, androgens, leptin and insulin) along with breastfeeding status at several time points postpartum in obese mothers may provide us with critical information. Animal studies also suggest that overfeeding during puberty may impair normal glandular development. A study evaluating the incidence of markers of insufficient glandular tissue/breast hypoplasia in obese mothers would be a potential first step in investigating a role for maternal obesity in impaired glandular development in humans. Given the impact of breastfeeding on both infant and maternal health, it is of critical importance that we focus future research on improving breastfeeding rates in this population.

## Conclusions

Obesity is a major risk factor for reduced initiation, duration and exclusivity of breastfeeding. We concluded that much of the evidence at this stage points to physiological factors such as difficult birth, delayed onset of lactogenesis II, and imbalances of hormones and adipokines as likely contributors to lower breastfeeding rates in obese women. Although we found four published breastfeeding interventions in this population, only a single intervention providing scheduled support by an IBCLC has shown a significant effect to increase breastfeeding duration and exclusivity in obese mothers. Further research is needed to identify modifiable behavioral and physiological variables that may lead to increased breastfeeding exclusivity and duration in obese mothers.

## Abbreviations

BMI: Body mass index
DOL: Delayed onset of lactogenesis II
PCOS: Polycystic ovarian syndrome
T4: Levothyroxine
TSH: Thyroid stimulating hormone
BIBS: Bassett improving breastfeeding studies
IBCLC: International Board Certified Lactation Consultant
